# Fetal electrocardiogram: ST waveform analysis in intrapartum surveillance

**DOI:** 10.1111/j.1471-0528.2007.01479.x

**Published:** 2007-10

**Authors:** I Amer-Wahlin, S Arulkumaran, H Hagberg, K Maršál, GHA Visser

**Affiliations:** a Section of Obstetrics, Department of Obstetrics and Gynaecology, Karolinska University Hospital Stockholm, Sweden; b Section of Obstetrics and Gynaecology, Division of Clinical Developmental Sciences, St George’s University of London London, UK; c Perinatal Center, Sahlgrenska Academy KK East Hospital, Gothenburg, Sweden; d Department of Obstetrics and Gynaecology, University Hospital Lund, Sweden; e University Medical Centre Utrecht Location WKZ Department of Obstetrics and Gynaecology Utrecht, the Netherlands

**Keywords:** Fetal ECG, guidelines, intrapartum surveillance, ST waveform analysis

## Abstract

ST waveform analysis of fetal electrocardiogram (ECG) for intrapartum surveillance (STAN) is a newly introduced method for fetal surveillance. The purpose of this commentary is to assist in the proper use of fetal ECG in combination with cardiotocography (CTG) during labour. Guidelines and recommendations concerning CTG and ST waveform interpretation and classification are stated that were agreed on by the European experts on ST waveform analysis for intrapartum surveillance during a meeting in Utretcht, the Netherlands in January 2007.

The technique was developed in Sweden and has widespread use in Scandinavia with the help of ‘expert’ centres, which help in education and training. It is used to a lesser extent over the rest of Europe and has recently been approved by the Food and Drug Administration for use in the USA.

With the introduction of this new technique, pitfalls, limitations and user errors have emerged with extended use. The two articles in this issue of the *BJOG*draw attention to these.^1^, ^2^ To resolve such issues arising from some cases in Scandinavia, a Nordic meeting took place in November 2006, and European problems were discussed during a European meeting in Utrecht, the Netherlands, in January 2007. A general agreement was reached that fetal heart rate (FHR) interpretation was still the main problem. Decision making with the STAN technology uses computer analysis of the electrocardiogram (ECG) with visual interpretation of the cardiotocogram (CTG). The expert group recommended slight revision of the STAN guidelines and measures to overcome user errors to reduce ambiguity and the risk of adverse outcome. This commentary outlines these recommendations.

## Prerequisites for initiation of STAN monitoring

A checklist was suggested to be used at the start of recording (Table 1). STAN calculates the initial reference baseline T/QRS using the first 20 T/QRS data recorded and resets the baseline if it becomes lower or after 3 hours of recording. A ST rise is detected when a sequence of T/QRS data are recorded, which significantly exceeds this T/QRS baseline, and an ST event is flagged by the computer. In a case of pre-existing fetal hypoxia, the ST change may have already taken place and further ST rise may not occur. Therefore, STAN recordings should start during the first stage of labour, with ideally a reassuring FHR trace.

**Table 1:** ST analysis checklist at start-up

**Before starting STAN**
•>36 + 0 gestational weeks
•Ruptured membranes
•No contraindication for scalp electrode
•First stage, no active or involuntary pushing at onset
**After start-up**
•Normal ECG waveform with sufficient signal quality
•Event log message baseline determined
•Check for reactivity and nondeteriorating fetal state at the onset of a STAN recording, classify FHR!

Based on a large prospective study where acid–base values were evaluated with CTG and ECG changes, the expert group considers it acceptable to start STAN monitoring on a nonreassuring CTG trace if the previous FHR pattern includes signs of reactivity (accelerations and/or FHR variability).^2^ Abnormal FHR at the start of recording without previous FHR information requires assessment of the fetal state prior to the application of STAN, for example analysis of fetal scalp pH and/or FHR reactivity with digital or vibroacoustic stimulation. Absence of ST events in a situation with nonreassuring FHR trace from the start could be related to previous compromise in a fetus that is unable to respond with ECG changes. If additional assessment of the fetal condition in such a case is not possible, the need for intervention should be based on FHR, clinical situation and fetal blood sampling (FBS) but not on STAN information.

## Signal quality

Fetal ECG ST analysis requires good signal quality. Continuous data (with at least one T/QRS ratio/minute) are needed to obtain reliable ST information. Gaps in the T/QRS ratios for more than 4 minutes may result in missed STAN events. This is particularly important in the presence of intermediary or abnormal FHR and during second stage when the condition of the fetus may deteriorate rapidly.

## Disconnection of ST waveform analysis

The reference T/QRS baseline calculated previously is kept when the machine is temporarily disconnected by using the ‘temporary end’ function. However, ST events that may occur during the disconnected period will not be detected. Therefore, pausing a recording when the FHR is abnormal is not recommended, as significant STAN events may be missed. If the FHR has become abnormal during temporary disconnection, the situation maybe similar to starting a recording with an abnormal trace, that is there is a need to check the fetal status using FBS or stimulation tests.

## FHR classification used with STAN technology

With the use of STAN, the FHR pattern is classified according to the International Federation of Obstetrics and Gynecology guidelines.^3^ With a combination of several intermediary observations, the FHR should be classified as abnormal (Table 2). Calling for additional expertise in assessing FHR should be considered an intervention and is as important as alleviation of the cause(s) of fetal compromise, such as stopping oxytocin infusion or repositioning of the mother.

**Table 2:** CTG classification

CTG-class	Baseline heart rate	Variability/reactivity	Decelerations
Normal CTG	•110–150 bpm	•5–25 bpm	•Early uniform decelerations;
		•Accelerations	•Uncomplicated variable decelerations with a duration of <60 sec and loss of <60 beats
Intermediary CTG	•100–110 bpm	•>25 bpm (saltatory pattern)	•Uncomplicated variable decelerations with a duration <60 sec and loss of >60 beats
	•150–170 bpm	•<5 bpm for >40 minutes with absence of accelerations	
	•Short bradycardia episode (<100 bpm for ≤3 minutes)		
**A combination of several intermediary observations will result in an abnormal CTG**			
Abnormal CTG	•150–170 bpm and reduced variability	•<5 bpm for >60 minutes	•Complicated variable decelerations with a duration of >60 sec
	•>170 bpm	•Sinusoidal pattern	
	•Persistent bradycardia (<100 bpm for >3 minutes)		•Repeated late uniform decelerations
Preterminal CTG	Total lack of variability (<2 bpm) and reactivity with or without decelerations or bradycardia		

bpm, beats per minutes.

## Gradual deterioration of the FHR pattern in the absence of ST events

In rare cases, the FHR pattern may gradually change from normal to abnormal, without the appearance of ST events (see paper by Westerhuis *et al.*in this issue).^1^ An abnormal FHR pattern for more than 60 minutes (or earlier if the CTG deteriorates rapidly) with normal ST requires qualified assessment and checking for nondeteriorating fetal state (with a preterminal FHR pattern, intervention is always indicated, irrespective of the ST data.

## Intervention should be undertaken according the STAN guidelines

Intervention depends on the cause of fetal compromise and the stage of labour. During the first stage, interventions may consist of intrauterine resuscitation (cessation of oxytocin infusion and/or acute tocolysis in cases of excessive uterine contractions), correction of maternal hypotension or amnioinfusion. A caesarean section is only one of the options. Recovery requires both CTG and ST to be normalised and a FBS may be required to verify recovery. During the second stage of labour with active pushing, immediate operative delivery is recommended, unless spontaneous delivery is to be anticipated in the next 5–10 minutes.

In the presence of STAN event and suspicious or abnormal CTG calling for intervention according to the STAN guidelines, this should be undertaken within 20 minutes (Table 3).

**Table 3:** STAN simplified clinical guidelines. Intervention recommended on the basis of CTG abnormalities and ST events*

	Intermediary CTG	Abnormal CTG	Preterminal CTG
Episodic T/QRS rise	>0.15	>0.10	Immediate delivery
Baseline T/QRS rise	>0.10	>0.05	
Biphasic ST	Three biphasic ST events	Two biphasic ST events	

* These guidelines are applicable to a term pregnancy of 36 completed gestational weeks or more and indicate situations in which intervention is required. This means calling for further expertise in assessing FHR data, alleviation of the cause(s) of fetal distress (overstimulation with oxytocin or maternal hypotension) or delivery. During the second stage of labour with active pushing, immediate operative delivery is recommended, unless spontaneous delivery is to be anticipated in the next 5–10 minutes.

## Maternal fever

STAN is a method for detection of acute intrapartum asphyxia not fetal infections. In the presence of maternal pyrexia, even intermediary FHR changes may be regarded as significant in connection with an ST event.

## Biphasic ST events

Biphasic (BP) ST events should be considered in relation to the window of recording. This implies that two BP events in combination with an abnormal CTG calls for intervention. The time-span between the BP should be related to the clinical situation. Appropriate and consistent classification of the FHR patterns is still the weakest link in intrapartum monitoring, as education and training in FHR interpretation is not as widespread as the method itself.

## Supplementary material

The following supplementary material is available for this article:

Appendix S1 Acknowledgements.

These materials are available as part of the online article from:

http://www.blackwell-synergy.com/doi/abs/10.1111/j.1471-0528.2007.01479.x

(This link will take you to the article abstract).
