# Supplementary material for: Fetal electrocardiogram: ST waveform analysis in intrapartum surveillance
Source: BJOG. 2007 Oct;114(10):1191–3. doi: 10.1111/j.1471-0528.2007.01479.x (PMC2239304; doi:10.1111/j.1471-0528.2007.01479.x)
Supplement: Appendix S1 — Acknowledgements. [file bjh0114-1191-SD1.doc]

**Appendix S1. Acknowledgements**

We acknowledge the valuable input of the members European STAN expert group in writing this paper : Diogo Ayres de Campo Porto Portugal, Diana Bach, Hvidovre, Denmark, Saskia Bijvoet, Amsterdam, Holland,, Vincent Cararac, Barcelona Spain, Jan Derks, Utrecht, Holland , Werner Diehl, Hamburg, Germany ,Alessandra Giannesi, Lyon France, Wilfried Gyselaers, Genk, Belgium , Zdenek Hajek, Prague Czech Republic, Myriam Hanssens, Leuven, Belgium Yves Jacquemyn, Antwerp Belgium, Anneke Kwee, Utrecht, Holland, Roberto Luzietti, Perugia, Italy , Hakan Noren, Göteborg Sweden, Nina Palmgren, Copenhagen, Denmark, Karl Rosen, Göteborg, Sweden, , Susanna Timonen, Turku, Finland,, Austin Ugwumadu, London UK, Herman Van Geijn, Amsterdam Holland ,Christophe Vayssiere, Strasbourg France ,Michelle Westerhuis, Utrecht, Holland, ,.Branca Yli, Oslo, Norway
